# Molecular Confirmation of Rocky Mountain Spotted Fever Epidemic Agent in Mexicali, Mexico

**DOI:** 10.3201/eid2409.171523

**Published:** 2018-09

**Authors:** Luis Tinoco-Gracia, Moisés Rodríguez Lomelí, Sawako Hori-Oshima, Nicole Stephenson, Janet Foley

**Affiliations:** Universidad Autónoma de Baja California, Mexicali, Mexico (L. Tinoco-Gracia, M. Rodríguez Lomelí, S. Hori-Oshima);; University of California, Davis, California, USA (N. Stephenson, J. Foley)

**Keywords:** emerging infectious disease, *Rhipicephalus sanguineus*, *Rickettsia rickettsii*, tick-borne disease, rickettsia, bacteria, ticks, Mexicali, Mexico, vector-borne infections

## Abstract

Since 2008, a large epidemic of Rocky Mountain spotted fever has been emerging among humans and dogs in Mexicali, adjacent to the United States in Baja California, Mexico. We molecularly confirmed the causative agent; this information can be used to study the origin and dynamics of the epidemic.

Rocky Mountain spotted fever (RMSF), caused by the bacteria *Rickettsia rickettsii*, is responsible for more human deaths than any other tickborne disease in North America (*1*). During 1999–2007, a total of 80 fatal cases were reported from Sonora, Mexico, alone (*2*). Recent epidemics in Arizona (USA) and Sonora have been associated with the brown dog tick (*Rhipicephalus sanguineus*) (*3**,**4*), whereas most cases in the United States have been transmitted by bites of infected *Dermacentor* spp. ticks (*5*). The risk to humans is heightened by the epidemiologic cycle of the brown dog tick, a cosmopolitan tick that prefers the dog as its host and can live its entire life cycle in a periurban setting, often spending its off-host time indoors. *R. sanguineus* ticks, in addition to being vectors of *R. rickettsii*, are probable or confirmed vectors of *Leishmania*, *Coxiella burnetii,* and *R. conorii* (*6*).

## The Study

In 2008, an epidemic of RMSF began in Mexicali, adjacent to the US border in Baja California, Mexico. In 2015, the Mexican Ministry of Health declared the epidemic an epidemiologic emergency, which as of 2018 has affected ≈4,000 persons. In 2014, a fatal human case in Imperial County, CA, USA, was probably associated with the Mexicali epidemic. Overall, since 2000, in the United States, the incidence of RMSF has reportedly increased ≈4-fold (*7*); this dramatic increase may be caused in part by increased transmission via the brown dog tick but also by changes in reporting and inclusion of false-positive test results in case diagnoses.

Local response to the ongoing epidemic in Mexicali has involved the Secretariat of Health and doctors and researchers at the Universidad Autónoma de Baja California schools of medicine and veterinary medicine. During 2008–2009, in the impoverished neighborhood of Los Santorales in Mexicali, at least 13 persons died of RMSF. Under agreement with the Sector Salud de Mexicali, the Universidad Autónoma de Baja California veterinary team documented 81% seroprevalence among local dogs and confirmed active *R. rickettsii* infection in a human resident by conducting PCR of kidney tissue (*8*). Of 120 persons from Mexicali with clinical signs compatible with RMSF, 30 were positive by PCR for the gene *gltA*, according to an unpublished method (*9*). In 2014, the local team partnered with researchers at the University of California, Davis (Davis, California, USA), to further molecularly characterize the strains of *R. rickettsii* and *R. sanguineus* ticks from Mexicali. We provide definitive molecular confirmation of the identity of the disease agent causing the Mexicali epidemic.

The University of California, Davis, laboratory received DNA extracted by use of QIAGEN Blood and Tissue Kits (Valencia, CA, USA) from 16 cases from Mexico. Initial *R. rickettsia–*specific real-time PCR for the citrate synthase gene (*10*) was positive for 10 samples. To obtain products for DNA sequencing, we performed traditional PCR for the *ompA* and *17kDa* genes as published (*11*,*12*). Sequence-confirmed positive DNA and water-containing negative control reactions were incorporated in each PCR run. Results were assessed by electrophoresis and UV-transillumination of 1% agarose gels stained with Gelstar (Lonza; Rockland, ME, USA). Bands of the expected size were excised and cleaned with a QIAquick Gel Extraction Kit (QIAGEN) according to the manufacturer’s instructions. Products were sequenced in the forward and reverse directions in an ABI Prism 3730 Genetic Analyzer at the ^UC^DNA Sequencing Facility at the University of California, Davis. Sequences were manually trimmed and corrected if the nucleotide could be unambiguously determined, then aligned by using CLC Main Workbench 6 (CLC bio, Waltham, MA, USA).

We successfully obtained *ompA* and *17kDa* products from 5 samples and compared the sequences with those in the GenBank database by using BLAST (http://blast.ncbi.nlm.nih.gov/Blast.cgi). For *ompA,* the resulting 472-bp amplicons from the 5 products from Mexicali were 100% similar. For this gene, numerous accessions in GenBank also have 100% homology with 100% coverage, including strains Sheila Smith, Hauke, Hilo, Colombia, and Arizona. Sequences of *17kDa* spanned 206 bps and were also completely homologous among them. This gene did not differentiate to species but was 100% homologous with *R. rickettsii*, *R. parkeri*, and others in the database. Representative sequences from Mexicali were submitted to GenBank (accession nos. KY689935 for *ompA* and KY824575 for *17kDa*).

Among sequence-confirmed samples, data were not available for 1 sample. The other 4 samples were collected in June, July, and September 2013 and April 2014. Two samples were from men (41 and 25 years of age) and 2 from women (18 and 29 years of age); all patients had dogs with ticks. Signs and symptoms were fever and headache for all; for 1 patient, a rash and convulsions also developed. The 2 men died and the 2 women survived with treatment. All patients had home addresses in various parts of Mexicali, including central west, southwest, and southeast bordering agricultural land. Clinical data were not available for patients for whom samples were considered PCR positive but not sequence confirmed, although inclusion of such clinical data and risk factors could bias interpretation if they were false positive or only weakly positive.

## Conclusions

The RMSF epidemic in Mexicali has not been contained and may be spreading to other parts of Baja California and into the United States. More data are needed before we can understand why this epidemic emerged, where the specific areas of high risk for exposure to infected ticks are located, and whether the particular *R. rickettsii* strain or relationship with this *R. sanguineus* tick strain is likely to be particularly invasive or virulent. Pockets of RMSF have occurred in Mexico since at least 1947, when cases attributable to the brown dog tick in Sonora, Sinaloa, Coahuila, and Durango were described (*13*). Given the very limited phylogeographic resolution available for *R. rickettsii* in many of the commonly used PCR products (*14*), it is not known whether the bacteria in the Mexicali epidemic originated from Sonora or more distantly. Next steps include obtaining a culture of the bacteria from Mexicali, studying bacterial virulence in vitro or in animal models, and assessing vector competence of the Mexicali *R. sanguineus* tick strain for *R. rickettsii.* Epidemiologic data on the spatial distribution and prevalence of infection in dogs are needed.

Aggressive intervention achieved partial and temporary resolution of the Arizona and Sonora epidemics, which were localized and relatively small; these interventions included dog spay and neuter programs, treatment of houses against ticks, and use of a long-acting tick collar (Seresto; Bayer, Shawnee Mission, KS, USA) directly on the dogs (*15*). However, the dog collars were initially donated and are prohibitively expensive and not feasible for the scope of the Mexicali epidemic. This large epidemic in a major city will require a far greater and more creative public health response. Studying this epidemic offers an opportunity to understand the origin and dynamics of this epidemic and can inform response to emerging tickborne diseases in general.

## References

[1] Biggs HM, Behravesh CB, Bradley KK, Dahlgren FS, Drexler NA, Dumler JS, et al. Diagnosis and management of tickborne rickettsial diseases: Rocky Mountain spotted fever and other spotted fever group rickettsioses, ehrlichioses, and anaplasmosis—United States. MMWR Recomm Rep. 2016;65(RR-1):1–44. 10.15585/mmwr.rr6502a1 27172113

[2] Álvarez-Hernández G, Roldán JFG, Milan NSH, Lash RR, Behravesh CB, Paddock CD. Rocky Mountain spotted fever in Mexico: past, present, and future. Lancet Infect Dis. 2017;17:e189–96. 10.1016/S1473-3099(17)30173-1 28365226

[3] Nicholson WL, Paddock CD, Demma L, Traeger M, Johnson B, Dickson J, et al. Rocky Mountain spotted fever in Arizona: documentation of heavy environmental infestations of *Rhipicephalus sanguineus* at an endemic site. Ann N Y Acad Sci. 2006;1078:338–41. 10.1196/annals.1374.065 17114735

[4] Alvarez-Hernández G. La Fiebre Manchada de las Montañas Rocosas, una epidemia olvidada. Salud Publica Mex. 2010;52:1–3. 10.1590/S0036-36342010000100002 20464246

[5] Regan JJ, Traeger MS, Humpherys D, Mahoney DL, Martinez M, Emerson GL, et al. Risk factors for fatal outcome from rocky mountain spotted Fever in a highly endemic area-Arizona, 2002-2011. Clin Infect Dis. 2015;60:1659–66. 10.1093/cid/civ116 25697742 PMC4706357

[6] Dantas-Torres F. Biology and ecology of the brown dog tick, *Rhipicephalus sanguineus.* Parasit Vectors. 2010;3:26. 10.1186/1756-3305-3-26 20377860 PMC2857863

[7] Centers for Disease Control and Prevention. Rocky Mountain spotted fever—statistics and epidemiology [cited 2017 Aug 1]. https://www.cdc.gov/rmsf/stats/index.html

[8] Tinoco L. Diagnostico serologico y molecular de un brote de riquetsiosis (*Rickettsia rickettsii*) en perros y un humano en la cuidad de Mexicali, B.C. XXXIV Congreso Nacional de Infectologia, México; 2009 Oct 7–10; Guadalajara, Jalisco State, Mexico.

[9] Gómez-Castellanos P, Tinoco-Gracia L, López-Valencia G, Oshima S, Medina Basulto G. Deteccion de especies del genero *Rickettsia* por PCR en pacientes del sector salud y analisis de factores de riesgo en Mexicali Baja California. Rev Biomed. 2015;26:59.

[10] Kato CY, Chung IH, Robinson LK, Austin AL, Dasch GA, Massung RF. Assessment of real-time PCR assay for detection of *Rickettsia* spp. and *Rickettsia rickettsii* in banked clinical samples. J Clin Microbiol. 2013;51:314–7. 10.1128/JCM.01723-12 23135935 PMC3536194

[11] Anstead CA, Chilton NB. A novel *Rickettsia* species detected in vole ticks (*Ixodes angustus*) from western Canada. Appl Environ Microbiol. 2013;79:7583–9. 10.1128/AEM.02286-13 24077705 PMC3837795

[12] Webb L, Carl M, Malloy DC, Dasch GA, Azad AF. Detection of murine typhus infection in fleas by using the polymerase chain reaction. J Clin Microbiol. 1990;28:530–4. 2108995 10.1128/jcm.28.3.530-534.1990 PMC269657

[13] Bustamante M, Varela G. Distribucion de las rickettsiasis en Mexico. Rev Inst Salubr Enferm Trop. 1947;8:3–13.

[14] Paddock CD, Denison AM, Lash RR, Liu L, Bollweg BC, Dahlgren FS, et al. Phylogeography of *Rickettsia rickettsii* genotypes associated with fatal Rocky Mountain spotted fever. Am J Trop Med Hyg. 2014;91:589–97. 10.4269/ajtmh.14-0146 24957541 PMC4155566

[15] Drexler N, Miller M, Gerding J, Todd S, Adams L, Dahlgren FS, et al. Community-based control of the brown dog tick in a region with high rates of Rocky Mountain spotted fever, 2012-2013. PLoS One. 2014;9:e112368. 10.1371/journal.pone.0112368 25479289 PMC4257530

